# Draft genome sequencing data of the bacterial wilt, *Ralstonia pseudosolanacearum* T2C-Rasto, from *Cucumis sativus*, in An Giang province, Mekong Delta - Southwest Vietnam

**DOI:** 10.1016/j.dib.2023.109252

**Published:** 2023-06-01

**Authors:** Thanh Binh Le, Minh Ngoc Truong, Ba Tho Nguyen, Dinh Quang Vo, Trang Thi Phuong Phan

**Affiliations:** a University of Science, Vietnam National University Ho Chi Minh City, Ho Chi Minh City, Viet Nam; b Vietnam National University, Ho Chi Minh City, Viet Nam; c National Center for Technological Progress – HCM Branch, Ho Chi Minh City, Viet Nam

**Keywords:** Agriculture, Bacterial wilt disease, Cucumis sativus, Draft genome sequencing, Ralstonia sp

## Abstract

*Ralstonia solanacearum* is one of the major plant pathogens causing bacterial wilt disease in a variety of plant species. In Vietnam, according to our knowledge, we first discovered *R. pseudosolanacearum,* which is one of four phylotypes of *R. solanacearum,* as a causal agent wilting in cucumber (*Cucumis sativus*). Due to the latent infection of *R. pseudosolanacearum* and its heterogenous species complex, controlling the disease becomes difficult.Therefore, the study of *R. pseudosolanacearum* has great significance to generate effective disease management and treatment. Here, we assembled the isolate *R. pseudosolanacearum* strain *T2C-Rasto,* which possessed 183 contigs with 67.03% GC content of 5,628,295 bp in. This assembly included 4,893 protein sequences, 52 tRNA genes, and 3 rRNA genes. In addition, the virulence genes involved in the colonization of the bacterium and wilting to the host were defined in twitching motility (*pilT, pilJ, pilH and pilG*)**,** chemotaxis (*cheA* and c*heW*), type VI secretion system (*ompA, hcp, paar, tssB, tssC, tssF, tssG, tssK, tssH, tssJ, tssL and tssM*), type III secretion system (*hrpB* and *hrpF*).

**Specifications Table**

**Table:** 

Subject	Biology
Specific subject area	Microbiology, Genomics, Biotechnology
Type of data	Genomic sequence, predicted genes and annotation
How the data were acquired	Whole genome sequencing using Illumina MiniSeq Sequencing
Data format	Raw and analysed
Description of data collection	*R. pseudosolanacearum* strains T2C-Rasto, isolated from wilt *Cucumis sativus*, in An Giang province, Mekong Delta - southwest Vietnam. The total DNA was extracted, followed by library preparation sequenced on Illumina MiniSeq platform. Reads were assembled and annotated.
Data source location	Town/City: Chau Thanh, An Giang Country: Vietnam Latitude and longitude for collected samples: 10°27′00.0"N 105°19′25.3"E
Data accessibility	Raw read is available in the SRA database under the Bioproject PRJNA748156 (https://www.ncbi.nlm.nih.gov/bioproject/PRJNA748156) and assembled draft genome sequence (.fasta) has been deposited at GenBank under the accession number JAHWRH010000000 (https://www.ncbi.nlm.nih.gov/nuccore/JAHWRH000000000). The annotation result and supplemental data are available here: https://data.mendeley.com/datasets/7whx24cxwg/draft?a=284df775-1bb0-4e48-b51a-5b6148062612

## Value of the Data

• Bacteria wilt caused by *R. pseudosolanacearum* strains T2C-Rasto is one of the most damaging diseases for cucumber production, its genetic characteristics will provide useful data for disease management and further examination on treatment.
• Data could be of interest from groups focus on the evaluation of bacterial antagonists of *Ralstonia solanacearum*.
• Data can serve future analyses of the relationship between microorganism profiles cause cucumber wilt and risks of disease outbreak due to antibiotic resistance genes spread.

## 1 Objective

Cucumber bacterial wilt caused by *R. solanacearum* in Vietnam has not been reported before. Moreover, the high genetic diversity of the *R. solanacearum* raises issues for assessing the outcome of the treatment [1,2], particularly biological control strategies and resistant varieties. Therefore, understanding the genetic information of *R. solanacearum* is necessary, such as virulence and resistance genes, to be able to devise an effective treatment and prevention measure. We hereby provide the first draft genome of *R. pseudosolanacearum* T2C-Rasto.

## 2 Data Description

**Fig. 1:**
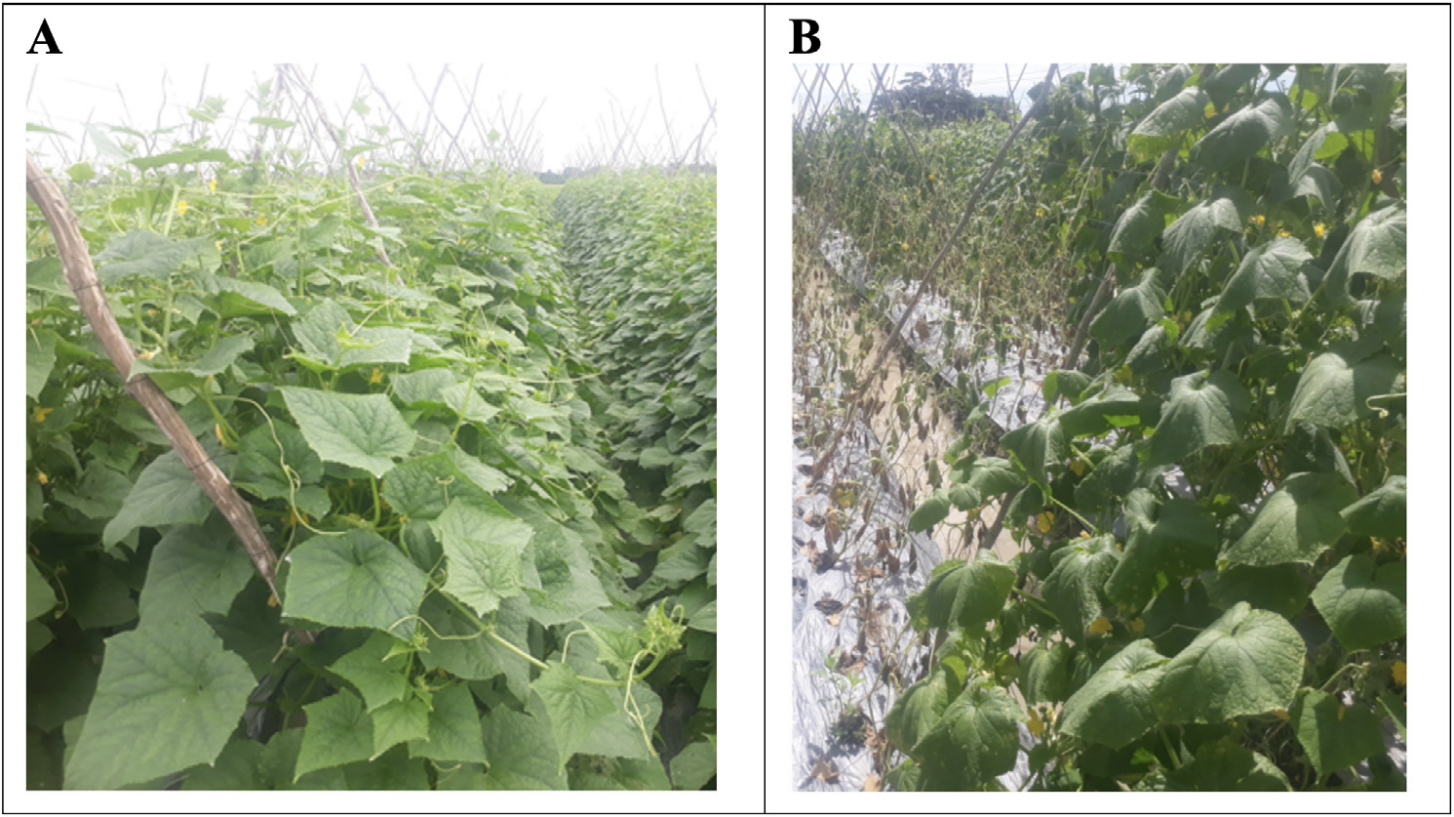
The pictures of non-infected (A) and infected (B) *Cucumis sativus.*

In Vietnam, *Cucumis sativus* is one of the largely consumed vegetables with the characteristic of being a short-term crop allowing farmers to grow multiple rounds of crops in a year, thus providing a stable income [3,4]. However, *C. sativus* wilt disease is caused by the bacterium, *R. solanacearum,* which has been threatening the huge losses of cucumber production. *R. solanacearum* is a heterogeneous species complex inducing postharvest disease known as foliage symptoms with rapid wilting of leaves and stems, particularly during the warmest season. Due to the latent infection of *R. solanacearum* during the preharvest stage, it becomes difficult to control the disease by chemical or physical treatment. The symptom onset appears on day 10 and then rapidly expands to entire the field within 5 days. Eventually, plants fail to recover, become yellow and brown necrotic and die following 3 days later (Fig. 1). Here, we present the draft genome sequences of *R. solanacearum* obtained from bacterial wilt diseased on *Cucumis sativus* in Vietnam.

**Fig. 2:**
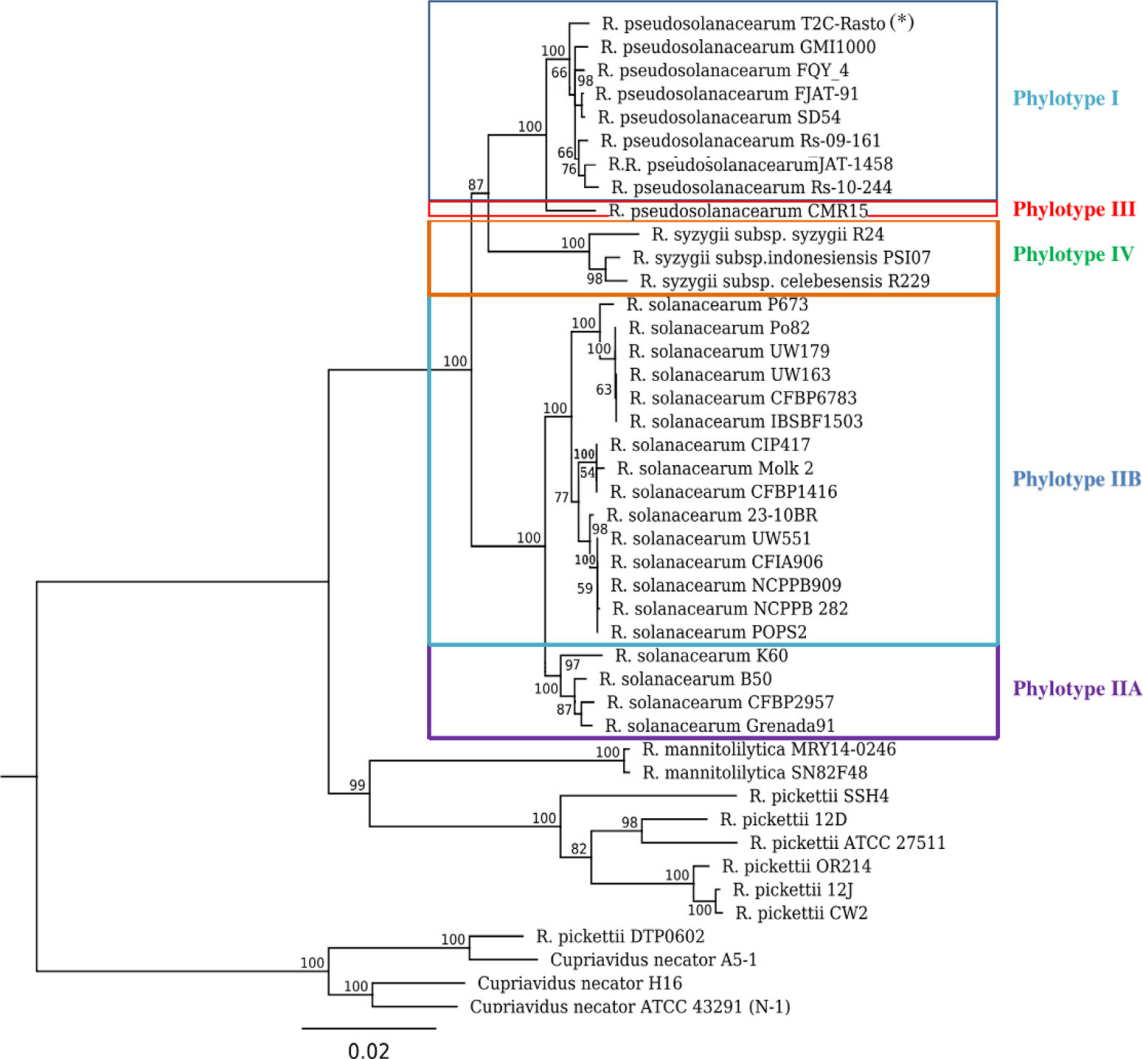
Neighbour-Joining phylogenetic trees based on concatenation of nucleotide acid sequences of *gdhA, mutS, adk, leuS, rplB and gyrB*[5] using the Tamura-Nei model, 1000 bootstrap replicates were computed. There were three strains of *Cupriavidus sp* used as an outgroup. Numbers above branches are bootstrap percentages.

The statistics of the sequencing and assembly data are presented in Table 1. The cleaned reads obtained by removing adapters and low-quality sequences were used for further analysis. The sequence of six housekeeping genes was used to create the phylogenetic tree (Fig. 2) and identify the phylotype of T2C-Rasto isolated [5]. The phylogenomic analysis demonstrated isolate T2C-Rasto was *R. pseudosolanacearum* in phylotype I found predominantly in Asia [6,7]. The draft genome sequence of isolate T2C-Rasto was 5,628,295 bp with an average GC content of 67.03% over 183 contigs at least length of 200bp. The total number of genes after annotation was 5,077, of which 4,893 were coding sequences, 3 rRNA and 52 tRNA genes.

**Table 1:** Genome features of *R. pseudosolanacearum T2C-Rasto.*

Attribute	*R. pseudosolanacearum T2C-Rasto* value
Number of raw paired end (PE) reads	8,145,376
Number of cleaned PE reads	7,904,919
Total number of cleaned bases	1,183,199,150
Length of the consensus sequence	5,628,295
GC content	67.03
Genome coverage	382
No. of contigs (>= 200 bp)	183
N50	105,684
Predicted coding genes	4,893
Number of tRNA genes	52
Number of 5S, 16S and 23S rRNA genes	1, 1, 1 (5S, 16S, 23S)
GenBank accession	JAHWRH000000000

In addition, the genome contained genes encoding the components of type VI secretion system (T6SS) involved in both host manipulation and interbacterial competition to prevent self-intoxication of the host [8], which are baseplates components (*tssA, tssF, tssG, tssK, tssJ, tssL* and *tssM*), the sheath components (*tssB, tssC*), T6SS effectors (*ompA, hcp and paar)* and accessory proteins (*tssH*)*.* The presence of *hrpB* and *hrpF* genes encoding the type III secretions systems (T3SS) were also detected, which enables a bacterium to deliver pathogenicity proteins into plant cells [9,10]. Furthermore, the genes identified for chemotaxis (*cheA* and *cheW*) and twitching motility (*pilT, pilJ, pilH and pilG*) were found in T2C-Rasto playing important role in locating and biofilm formation [11,12].

**Fig. 3:**
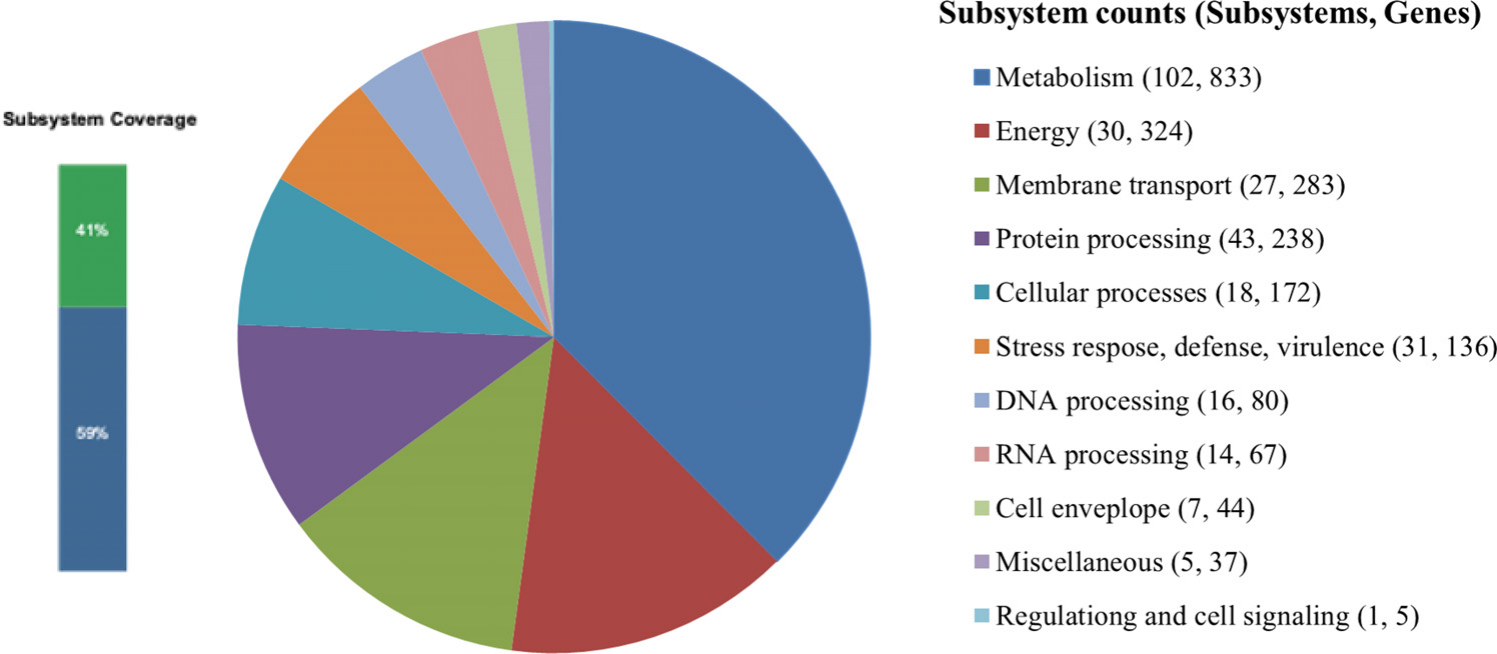
Distribution predicted genes of *R. pseudosolanacearum T2C-Rasto* assigned to subsystem categories. The subsystem coverage bar chart depicts the percentage of features found in PATRIC subsystem (green color) and features not assigned to a subsystem (blue color).

Annotation with the PATRIC server [13] revealed 294 subsystems. An overview of the distribution genes assigned to subsystem categories for the draft genome sequence of *R. pseudosolanacearum* strain T2C-Rasto generated by PATRIC is shown in Fig. 3.

## 3 Experimental Design, Materials and Methods

### 3.1 Sample Preparation and Genome Sequencing

Cucumber stem samples collected at the cucumber garden have just begun to suffer from wilt disease in An Giang province, Vietnam. The pure strain was isolated from the milky-white suspension found inside the wilt Cucumber stem and cultured on TZCA medium at 28°C for 48 hours. Through subsequent biochemical identification (Supplemental File 1), and 16S rRNA gene sequence analysis, we determined four strains belong to *Enterobacter sp, Stenotrophomonas sp, Paenibacillus sp, Ralstonia sp* (Supplemental File 2-5)*.*The challenge infection with *Ralstonia sp* strain on cucumber plants under greenhouse conditions revealed that *Ralstonia sp* T2C-Rasto has the strongest ability to cause wilt disease on cucumbers with 100% death rate on day 10. Therefore, *Ralstonia sp* strain T2C-Rasto was selected for further genomic analysis. Genomic DNA was extracted using a QIAamp DNA Mini Kit (Qiagen, Germany) and concentration was determined using a Qubit 4.0 Fluorometer (Invitrogen, USA). Sequencing libraries were constructed using the NEBNext® Ultra™ II DNA Library Prep Kit with a sequencing length of 2 × 150 bp (paired-end read sequencing) and evaluated by capillary electrophoresis (Bioanalyzer, Agilent) (Supplemental File 1). Whole genome sequence was performed using Illumina MiniSeq platform with V3 chemistry (Illumina, San Diego, CA).

### 3.2 Genome Assembly and Annotation

The raw reads were quality controlled using FastQC (version 0.11.5 (https://www.bioinformatics.babraham.ac.uk/projects/fastqc/). Trimmomatic (version 0.36) [14] was used to trim and remove the adapter sequence using the following parameters ILLUMINACLIP:TruSeq3-PE-2.fa:2:30:10 LEADING:3 TRAILING:3 SLIDINGWINDOW:4:15 MINLEN:36. To merge the trimmed reads, the BBMerge [15] from bbtools software suite was used and assembled using Unicycler (version 0.4.4) [16], with default settings. The genome statistics and annotation of the *R. pseudosolanacearum* T2C-Rasto were determined by using NCBI Prokaryotic Genomes Automatic Annotation Pipeline [17] and PATRIC [13]. The phylogenetic tree was generated based on the six house-keeping genes (*gdhA, mutS, adk, leuS, rplB* and *gyrB*) [5] and other reference genomes downloaded from NCBI and constructed in MEGA 6 using the Tamura-Nei model with 1000 bootstraps. The sequencing depth was computed by in-house script and report based on the depth of coverage of longest contig.

## Ethics Statements

This work did not involve human subjects, animal experiments and data collected from social media platforms.

## CRediT authorship contribution statement

**Thanh Binh Le:** Conceptualization, Investigation, Writing – original draft. **Minh Ngoc Truong:** Investigation. **Ba Tho Nguyen:** Investigation, Resources. **Dinh Quang Vo:** Data curation, Visualization. **Trang Thi Phuong Phan:** Conceptualization, Writing – review & editing.

## Declaration of Competing Interest

The authors declare that they have no known competing financial interests or personal relationships that could have appeared to influence the work reported in this paper.

## Data Availability

T2C-Ralsto Genome (Original data) (Mendeley Data).

## Appendix Supplementary materials

Image, application 1
