# Supplementary material for: Draft genome sequencing data of the bacterial wilt, Ralstonia pseudosolanacearum T2C-Rasto, from Cucumis sativus, in An Giang province, Mekong Delta - Southwest Vietnam
Source: Data Brief. 2023 Jun 1;48:109252. doi: 10.1016/j.dib.2023.109252 (PMC10293977; doi:10.1016/j.dib.2023.109252)
Supplement: Supplementary file 1 [file mmc1.docx]

**SUPPLEMENTARY**

**Figure S1: The biochemical tests were conducted on two isolation groups that were cultured on TZCA medium.** A. Catalase reaction, B. Oxidase reaction, C. KOH reaction, D. Lipase reaction. These isolates were gram-negative rod-shaped bacteria that produced mucilage with 3% KOH test and gas in the catalase reaction. Both groups were oxidase positive, as indicated by a blue-violet color change and the formation of a cloudy precipitate around the inoculum.

| 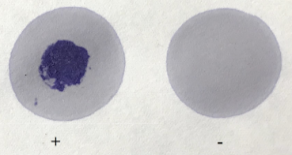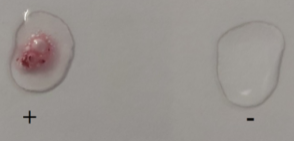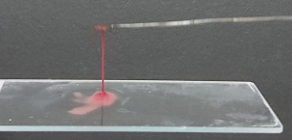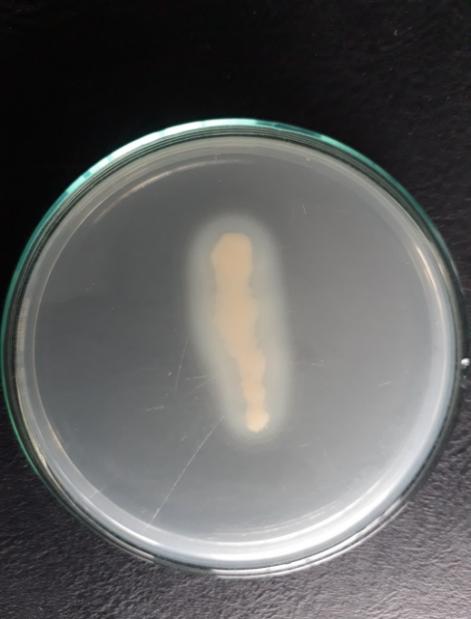 AC  B  C  D |
| --- |

**Figure S2: The pictures show infected (A) and non-infected (B) *Cucumis sativus* after 5 days of being challenged under greenhouse conditions.**


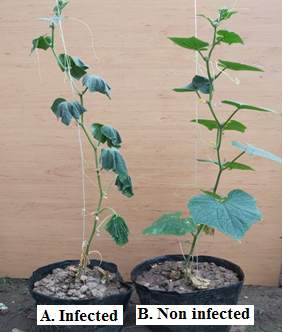


**Figure S3: The size of library was evaluated using Bioanalyzer**

*
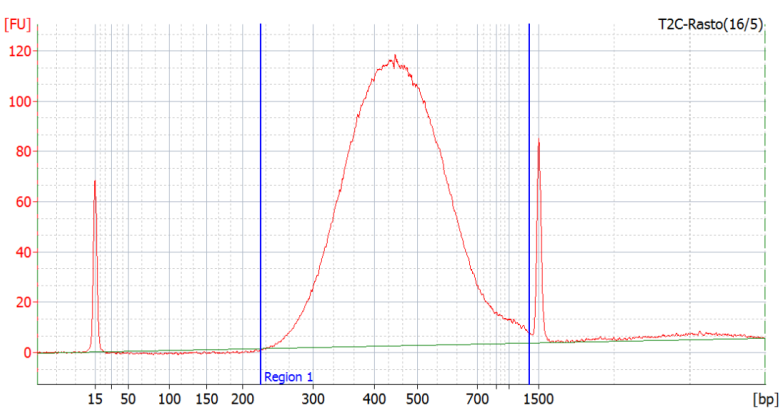
*
